# A Combination Treatment Strategy for Hemorrhagic Shock in a Rat Model Modulates Autophagy

**DOI:** 10.3389/fmed.2019.00281

**Published:** 2019-12-17

**Authors:** Xiaogang Chu, Richard Schwartz, Michael P. Diamond, Raghavan Pillai Raju

**Affiliations:** ^1^Department of Pharmacology and Toxicology, Medical College of Georgia, Augusta University, Augusta, GA, United States; ^2^Emergency Medicine, Medical College of Georgia, Augusta University, Augusta, GA, United States; ^3^Department of Obstetrics and Gynaecology, Medical College of Georgia, Augusta University, Augusta, GA, United States

**Keywords:** trauma, hemorrhage, shock, autophagy, mitochondria

## Abstract

Hemorrhagic shock leads to whole body hypoxia and nutrient deprivation resulting in organ dysfunction and mortality. Previous studies demonstrated that resveratrol, dichloroacetate, and niacin improve organ function and survival in rats following hemorrhagic shock injury (HI). We hypothesized that a combinatorial formula that collectively promotes survival will decrease the dose of individual compounds toward effective therapy for HI. Sprague-Dawley rats were subjected to HI by withdrawing 60% blood volume. NiDaR (Niacin-Dichloroacetate-Resveratrol; 2 mg/kg dose of each) or vehicle was administered following the shock in the absence of fluid resuscitation, and survival monitored. In order to study alterations in molecular mediators, separate groups of rats were administered NiDaR or vehicle along with resuscitation fluid, following HI. We observed significant improvement (*p* < 0.05) in survival following HI in animals that received NiDaR, in the absence of fluid resuscitation. In NiDaR treated animals that received resuscitation fluid, MAP was significantly increased compared to Veh-treated rats. HI-induced increase in systemic IL-6 levels and tissue expression of IL-6, IL-10, IL-1β, and IL-18 genes in the heart were attenuated with NiDaR treatment. NiDaR promoted autophagy following HI as demonstrated by reduced p-mTOR, increased p-ULK1 and p-Beclin. The combinatorial formula, NiDaR, reduced inflammation, promoted autophagy, and reduced doses of individual compounds used, and may be more effective in genetically heterogeneous population. In conclusion our experiments demonstrated that the combinatorial drug treatment has salutary effect in rats following HI.

## Introduction

Trauma is the leading cause of death in the 1–45 years age group (1, 2). Hemorrhage is a common cause of death following traumatic injuries (1–3). There is a lack of consensus on the use of any specific resuscitation strategy or adjuncts to fluid resuscitation following severe hemorrhage and therefore there is a need to develop suitable methods in the treatment of hemorrhagic shock (4). The availability of adjuncts that complement limited or no resuscitation may play critical role in prolonging life following hemorrhagic shock.

Our experiments using a rat model of hemorrhagic shock injury (HI) demonstrated that in the absence of volume replacement, a protracted metabolic homeostasis may be achieved using agents that potentiate cellular energetics (5–7). Cellular energetics is an important determining factor in maintaining homeostatic balance (7–9). Our studies unequivocally showed that the naturally occurring SIRT1 activator, resveratrol (RSV), can prolong survival in experimental rats following HI in the absence of fluid resuscitation (5). SIRT1 deacetylates target proteins in a NAD^+^ dependent mechanism. The effect of resveratrol in improving organ function in experimental animals subjected to hemorrhagic shock has been described by many laboratories (10–14).

We also observed prolonged survival following HI when the rats were treated with niacin (15). Niacin metabolizes to produce NAD^+^, increases SIRT1 activity and enhance mitochondrial function (16). Our continuing experiments seeking a role for cellular energetics in modulating outcome following HI demonstrated the significance of activation of pyruvate dehydrogenase (Pdh), a key protein connecting glycolysis and citric acid cycle in HI. We found that dichloroacetate (DCA), a classic inhibitor of pyruvate dehydrogenase kinase (Pdk), significantly improved organ function and survival following HI (6). Pdk is activated in tissue hypoxia resulting in inhibition of Pdh thereby reducing the availability of acetyl CoA to citric acid cycle. Therefore, inhibition of Pdk by DCA activates Pdh and citric acid cycle with resultant increase in ATP production.

The three compounds, resveratrol, DCA and niacin, agents that are known to enhance autophagy and mitochondria function (5, 6, 15, 17–22), were found to have optimal effect when used at a dose of 10–25 mg/Kg body weight in the rats. We hypothesized that a combinatorial formula that collectively promotes survival will decrease the dose of individual compounds toward improving survival after HI. In this study we tested whether a combination of resveratrol, DCA and niacin (NiDaR) can be used in reduced individual doses with similar outcome, and examined the regulation of autophagy following treatment with NiDaR.

## Materials and Methods

### Hemorrhage Injury Procedure

The animals were subjected to sham or HI procedure as described before (5, 6). Briefly, the animals were anesthetized with isoflurane (Henry Schein, Dublin, OH, USA) and a midline laparotomy (3 cm) was performed to induce soft tissue trauma. Femoral arteries and one femoral vein were cannulated (PE-50 tubing), for bleeding, blood pressure monitoring, and fluid resuscitation. Surgical sites were bathed with bupivacaine. Sham animals did not undergo hemorrhage or fluid resuscitation. Hemorrhagic shock injury was induced by bleeding rapidly to a MAP of 40 ± 5 mmHg in the first 10 min and bleeding continued for 45 min to remove 60% of the total blood volume. The animals were maintained in the state of shock by maintaining the low MAP for another 45 min. Two models were used. Model 1: In this model animals were resuscitated with fluid. The fluid resuscitation was carried out with Ringer lactate (RL; twice the volume of shed blood) following hemorrhage and shock, and lasted for 1 h. Vehicle (DMSO; 120 μL/rat) or NiDaR was administered at 10 min from the start of resuscitation. The animals were euthanized at 2 h after the end of fluid resuscitation and tissues saved. Model 2: Animals in this group were not resuscitated with fluid. This model was used for short-term survival studies to determine the effect of NiDaR on length of survival in the absence of fluid resuscitation. NiDaR or DMSO was administered (100 μL with 300–400 μl RL to flush the catheter) intravenously at the end of the shock period and survival observed. Euthanasia was performed by overdose of isoflurane followed by removal of heart.

### Immunoblotting Analysis

Immunoblotting procedures were performed as we described previously. Briefly, the heart samples were homogenized in the lysis buffer. Lysates were clarified at 12,000 g for 10 min at 4°C, and protein concentrations were determined by the Bradford protein assay (Bio-Rad Laboratories, Hercules, CA). Equal amounts of protein were loaded onto 8–12% SDS–PAGE, transferred onto polyvinylidene difluoride membranes, probed with the indicated primary antibody and the appropriate secondary antibody conjugated with horseradish peroxidase (Cell Signaling, Danvers, MA), and the immune complexes were detected by standard methods. The antibodies used in this study were as follows: GAPDH (Cell Signaling, Danvers, MA), p-P65 (Cell Signaling, Danvers, MA), P65 (Cell Signaling, Danvers, MA), p-ULK1 (Cell Signaling, Danvers, MA), ULK1 (Cell Signaling, Danvers, MA), p-Beclin1 (Cell Signaling, Danvers, MA), Beclin1 (Cell Signaling, Danvers, MA), LC3 (Cell Signaling, Danvers, MA), p-MTOR (Cell Signaling, Danvers, MA), MTOR (Cell Signaling, Danvers, MA), NLRP3 (Abcam, Cambridge, MA), SQSTM1 (Abcam, Cambridge, MA). HRP linked anti-rabbit or anti–mouse IgG second antibodies (Cell Signaling, Danvers, MA).

### Real-Time Polymerase Chain Reaction

The heart cytokines were tested by real time PCR. Total RNA was isolated using TRIZOL reagent (Thermo Fisher, Waltham, MA) and cDNA was synthesized using ImProm-II™ Reverse Transcription System (Promega, Madison, WI). Realtime PCR was performed as described previously. The sequences of the primers used were: IL-1β: Forward: CCCTGCAGCTGGAGAGTGTGG, Reverse: TGTGCTCTGCTTGAGAGGTGCT, IL-18: Forward: CAGACCACTTTGGCAGACTTCACT, Reverse: GGATTCGTTGGCTGTTCGGTCG, IL-6: Forward: GAGCCCACCAGGAACGAAA, Reverse: AACTGGCTGGAAGTCTCTTGC; IL-10: Forward: TGCGACGCTGTCATCGATTT, Reverse: GTAGATGCCGGGTGGTTCAA; β-actin: Forward: AGTACCCCATTGAACACG; Reverse: AATGCCAGTGGTACGACC. The results are expressed after normalizing to the values obtained for samples in control group.

### Lactate Assay

Heparinized blood samples were obtained prior to euthanasia. The plasma was separated and lactate levels were measured using a Lactate Assay Kit (Sigma, St. Louis, MO) according to the manufacturer's protocol.

### IL-6 Assay

Plasma IL-6 concentrations were determined using an IL-6 Rat ELISA Kit (Thermo Fisher, Waltham, MA) according to the manufacturer's protocol.

### Statistics

Data are presented as mean ± S.E.M. Student *t*-test or One-way ANOVA, was used for statistical analysis using Prism 6 (GraphPad Software). *p* < 0.05 was considered to be statistically significant. The primary outcome in the non-resuscitation study was survival duration. When MAP reduced to below 30 mm Hg the animals were euthanized, as death could not be an endpoint (6).

## Results

### Survival Study Using Combination Dose

**Figure 1:**
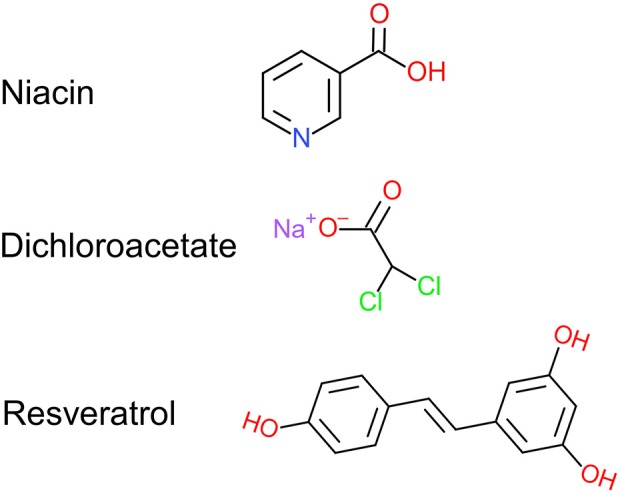
Chemical structure of niacin, dichloroacetate, and resveratrol. NiDaR is a combinatorial formulation of these three compounds. Drawn using Chemdoodle 2D sketcher.

**Figure 2:**
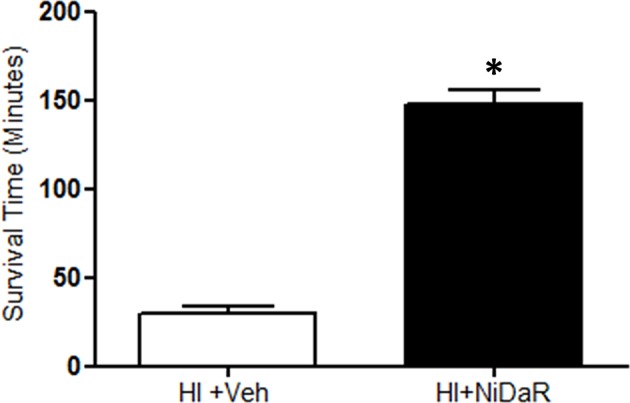
NiDaR prolongs life after hemorrhagic shock. Mean survival time (minutes) following HI and treatment in each of the experimental groups (mean ± SEM); *n* = 5–6 in each group, *indicates *p* < 0.05 compared to HI + Veh. NiDaR (2 mg/Kg of each constituent) was administered immediately following shock period.

In this study we tested whether reduced doses of Niacin, Dichloroacetate, and Resveratrol (NiDaR) administered as a combination can improve the outcome following HI (Figure 1). Each of the constituents in NiDaR was used at 2 mg/Kg dose. The mixture was administered intravenously either at 10 min after the start of fluid resuscitation or at the end of shock period in the subset of animals that did not receive fluid resuscitation. As shown in Figure 2, the combination dose significantly improved survival in the absence of fluid resuscitation. The animals were euthanized when the MAP reached below 30 mmHg as death cannot be an endpoint. It may be noted that when fluid resuscitation is not performed following hemorrhagic shock, in our model, MAP reaching 30 mmHg has been observed to be a point of no return for the rats.

### The Effect of NiDaR Treatment on MAP and Blood Lactate

**Figure 3:**
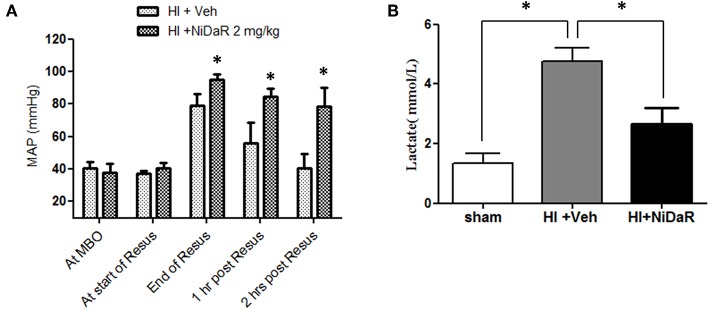
Effect of NiDaR on MAP and plasma lactate following hemorrhagic shock. **(A)** MAP following HI and treatment with NiDaR at maximum bleed out (MBO), start of resuscitation, end of resuscitation, 1 and 2 h post resuscitation. Bars represent mean ± SEM. *p < 0.05 vs. HI + Veh. **(B)** Plasma lactate levels in sham, HI + Veh, HI + NiDaR treatment groups; *n* = 5–6 in each group; bars represent mean ± SEM; *indicates *p* < 0.05.

As shown in Figure 3A, MAP was significantly improved in rats that received the combination dose as compared to the vehicle treated animals. A significant improvement was observed at the end of fluid resuscitation, as well as at 1 and 2 h after the end of resuscitation. As expected, blood lactate levels were significantly increased after HI in vehicle treated groups and decreased in NiDaR treated animals compared to veh group (Figure 3B).

### NiDaR Reduces Inflammation Following HI

**Figure 4:**
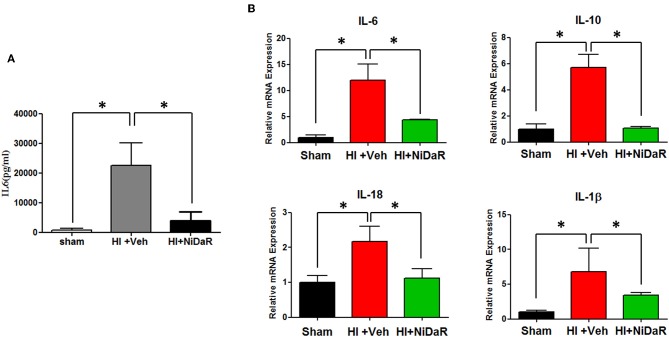
NiDaR attenuates inflammatory response following hemorrhagic shock. **(A)** Plasma IL-6 levels measured by ELISA. **(B)** Cytokine gene expression was measured by realtime PCR in the heart. *n* = 5–6 in each group; results include 2 technical replicates; bars represent mean ± SEM; *Indicates *p* < 0.05.

**Figure 5:**
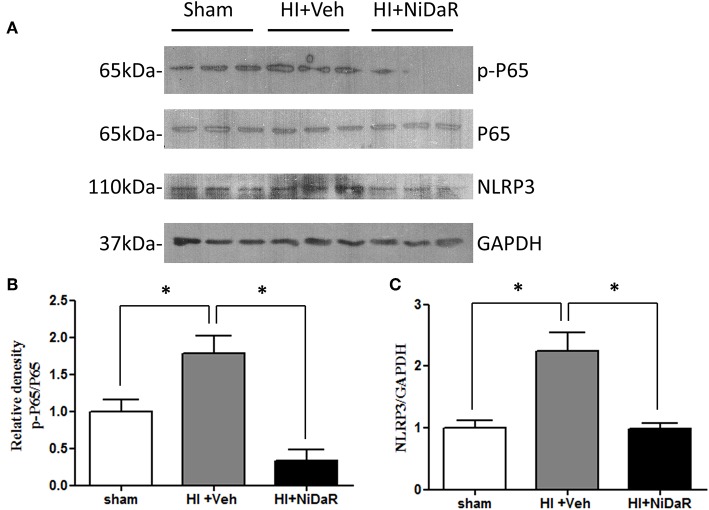
NiDaR reduces NF-κb and NLRP3 levels following hemorrhagic shock. **(A)** Representative immunoblotting of NF-κB P65, p-P65, NLRP3 in heart tissues of sham, HI and NiDaR treated rats. GAPDH was used as the loading control. **(B)** NF-κB p-P65, P65 intensities were quantified using Image J software(NIH). **(C)** NLRP3 and GAPDH intensities were quantified using Image J software (NIH). *n* = 6 animals each group, 2–3 technical replicates. *Indicates *p* < 0.05.

Furthermore, NIDaR treatment showed a significant effect on plasma IL-6 levels, the levels were markedly elevated in the untreated group whereas the combination dose was effective in reducing IL-6 in the plasma (Figure 4A). The expression of a number cytokine genes were tested in the heart tissue isolated from the rats subjected to Sham or HI. The expression of cytokines genes IL-6, IL-10, IL-18, and IL-1β were significantly elevated in the heart of rats subjected to HI, and were significantly decreased in NiDaR treated group compared to the Veh treated group (Figure 4B). Consistent with the reduction of inflammatory markers following HI, the phosphorylated NF-kb P65 subunit as well as NLRP3 showed a significant reduction after HI in NiDaR treated animals (Figures 5A–C).

### Effect of NiDaR Treatment on Autophagy

**Figure 6:**
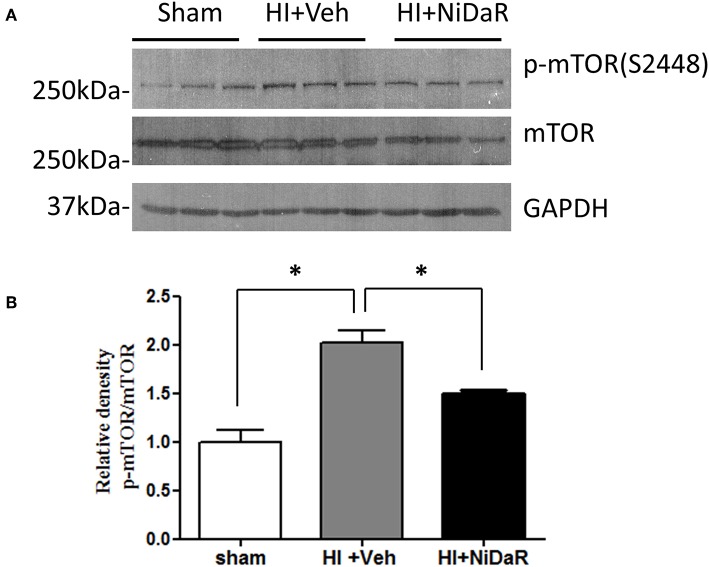
NiDaR treatment reduces p-mTOR in the heart after hemorrhagic shock. **(A)** Representative immunoblotting of p-mTOR (S2448) and mTOR in sham, HI and NiDaR treated rat heart tissues. GAPDH was used as the loading control. **(B)** p-mTOR(S2448) and mTOR intensities were quantified using Image J software(NIH). *n* = 6 animals in each group; 2-3 technical replicates. *Indicates *p* < 0.05.

**Figure 7:**
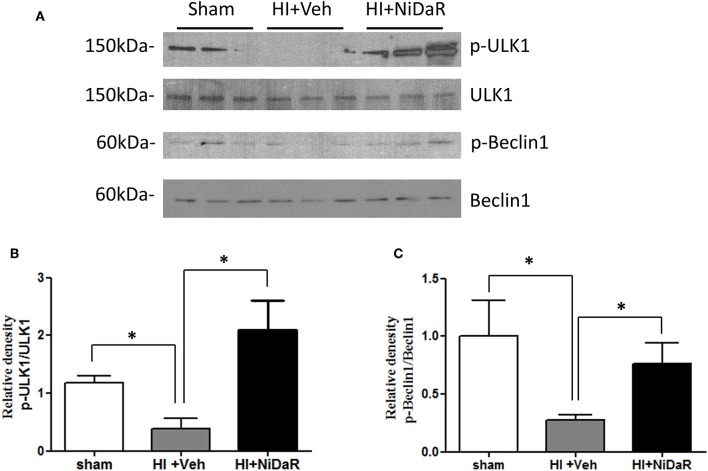
NiDaR promotes autophagy: effect on ULK1 and beclin. **(A)** Representative Immunoblotting of p-ULK1, ULK1, p-Beclin1, and Beclin1 in rat heart tissues. **(B)** *p*-ULK1 and ULK1 intensities were quantified using Image J software (NIH), *Indicates *p* < 0.05. *n* = 6. **(C)** p-Beclin1 and Beclin1 intensities were quantified using Image J software (NIH). *n* = 6 animals in each group; 2–3 technical replicates. *Indicates *p* < 0.05.

**Figure 8:**
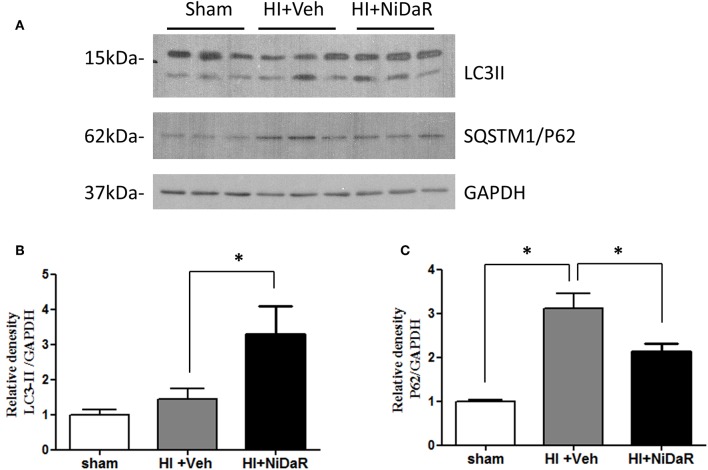
NiDaR promotes autophagy: effect on LC3-II and P62. **(A)** Representative Immunoblotting of LC3a/b and SQSTM1/P62 in rat heart tissues. GAPDH was used as the loading control. **(B)** LC3II and GAPDH intensities were quantified using Image J software (NIH), *Indicates *p* < 0.05. **(C)** P62 and GAPDH intensities were quantified using Image J software (NIH), There were 6 animals in each group, 2–3 technical replicates. *Indicates *p* < 0.05.

In order to understand the mechanism of NiDaR induced salutary effect, we investigated the effect of NiDaR on autophagy. HI was followed by activation of mTOR and NiDaR attenuated mTOR activation, mTOR inhibits autophagy (Figure 6). Furthermore, HI resulted in a significant reduction in the phosphorylated fraction of ULK, a constituent of Atg1 complex and NiDaR treated rats showed significantly increased p-ULK levels in the heart (Figure 7). Consistent with the increase of p-ULK, a similar increase was also observed for p-beclin suggesting that NiDaR can increase autophagy process after hemorrhagic shock. Interestingly, LC3 II (A/B) expression did not show significant change in the vehicle treated heart, but P62 expression was increased several fold after HI indicating accumulation of autophagy substrate with hemorrhagic shock (Figure 8). Whereas, LC3 II expression continued to increase after treatment in NiDaR treated rats, P62 expression demonstrated a significant decrease with the treatment demonstrating activation of autophagy by the combination dose.

## Discussion

We recently reported in separate papers that niacin, dichloroacetate, and resveratrol have salutary effect in hemorrhagic shock when administered individually at 10–25 mg/Kg dose each (5, 6, 15). All three compounds directly or indirectly potentiate mitochondrial function and autophagy. In this study we combined all the three compounds to activate multiple pathways and reduce the dose of individual constituents. Furthermore, clinically, such combination doses may be more protective in genetically heterogeneous population. We now demonstrate that when resveratrol, niacin, and dichloroacetate (NiDaR) were combined as a single treatment dose, effective dose of each of these compounds is decreased at least 5-fold. At this lower dose, NiDaR showed significant improvement in survival, decreased systemic and organ-specific inflammation and enhanced autophagic process. In previous studies 1 mg/Kg dose of individual compounds did not show significant survival benefit. The effectiveness of NiDaR was also associated with improved MAP and plasma lactate levels. The use of lower doses of individual compounds may result in reduced side-effects if any, and better effectiveness in a genetically diverse population, in a clinical setting.

A sudden onset of inflammation is a hallmark of hemorrhagic shock (23–26). A significant increase in the level of pro and anti-inflammatory cytokines are observed systemically as well as in several critical organs (27). Administration of NiDaR as a single dose intravenously along with the resuscitation fluid attenuated the inflammatory response. The IL-6 levels in the plasma as assessed by ELISA was significantly decreased in the animals that received NiDaR. Similarly, the gene expression of IL-6, IL-1β, IL-18, and IL-10 in the heart was also attenuated with the combination dose treatment demonstrating effectiveness of the therapy.

A number of immune and metabolic pathways have been previously investigated in hemorrhagic shock by our laboratory and others, and interventions have been proposed based on their effectiveness to control inflammation, improve cellular energetics or reduce cell death (12, 25, 28, 29). Our experiments in this study demonstrate that the cell maintenance process called autophagy is significantly activated in the heart when the animals subjected to HI were treated with NiDaR. Following HI, there was a significant decline in the phosphorylated form of ULK1 and beclin1 indicating suppression of autophagic process following HI. The nutrient sensor AMPK phosphorylates and activate ULK1 to promote autophagy (30). In a previous study we observed a decrease in the phosphorylation of AMPK following hemorrhagic shock and resuscitation (6). It is well-recognized that AMPK phosphorylation precedes an energy deprived state, however it is likely that acute injury with a sustained and prolonged reduction in ATP generation and mitochondrial function, as seen in hemorrhagic shock model, leads to reduced phospho-AMPK. Nevertheless, treatment of rats subjected to HI with a mitochondrial targeted agent, dichloroacetate, increased the p-AMPK/AMPK ratio in the heart, after HI, though the mechanism is unclear (6). ULK1 is an essential component of the Atg1 complex and phosphorylation is critical to its activity (30, 31). The significant decline in p-ULK1 observed following HI may be concomittant with the reduced p-AMPK levels thereby inhibiting an effective Atg1 complex formation. The increase in p-ULK1 after treatment with NiDaR indicates enhanced autophagy leading to improved survival. The activation of ULK1 by phosphorylation is a pre-requisite to initiate autophagy (32). ULK1 directly phosphorylates beclin 1 which is an important constituent of VSP34 complex (33), other entities in the complex are also regulated by ULK1 by direct phosphorylation (31). Our studies show a decrease in p-beclin1 in HI, along with the reduced p-ULK1 levels indicating a decrease in autophagic process with HI. Furthermore, treatment with NiDaR promoted autophagy as indicated by decreased p-mTOR, and increase in phosphorylated forms of both ULK1 and Beclin1 in the heart of groups of rats treated with NiDaR.

A decrease in the level of any of the components involved in Atg1 complex results in reduced autophagic clearance and accumulation of autophagic substrates such as P62 (34). The protein level of P62 in the heart was found to increase about 3-fold following HI and this may indicate a disrupted autophagy. However, treatment with the combination dose demonstrated a significant decrease in P62 levels demonstrating acceleration of autophagy. The LC3-II levels after HI remained the same as basal level indicating no significant increase in autophagic activity at 2 h after resuscitation, whereas the levels were significantly increased following NiDaR treatment, an indication of increased formation of autophagosomes. Nevertheless, the significant increase of LC3-II in the animals that were treated with the combination dose may indicate either an increased autophagy or inhibition of autophagosome degradation as LC3-II amount at a given time point does not necessarily estimate the autophagic activity (34). The latter cause may be ruled out considering the significant decrease in autophagic susbtrate (P62) with NiDaR treatment. However, the NiDaR group showed larger changes than maintaining levels observed in shams, it is not clear whether this is a dose effect or whether there could be consequences of such alterations in the treatment group.

One of the limitations of the study was that we administered only a single dose of NiDaR after hemorrhagic shock. The effect of administering NiDaR at multiple intervals or continuous infusion as was done in the clinical trial (CRASH-2) of tranexamic acid (35) needs to determined. In order to transition to clinically relevant studies, it is also necessary to test the effectiveness of NiDaR in long term survival with fluid resuscitation. Small volume treatments such as NiDaR can be given as first line treatment before resuscitation fluid could be available. The survival study in which we did not perform fluid resuscitation addresses this aspect.

Our experiments demonstrate lack of enhanced autophagic activity or inhibition of autophagy with HI and promotion of autophagic activity when treated with NiDaR. The positive regulation of autophagy by NiDaR in HI is not surprising as niacin, dichloroactetate, and resveratrol have been shown to independently activate autophagy in other models (17, 19, 36), however this is the first systematic demonstration of autophagic regulation following HI. In summary, the study shows that NiDaR attenuates inflammation and promote autophagy following hemorrhagic shock and this combinatorial formulation is promising in the treatment of hemorrhagic shock.

## Data Availability Statement

The datasets generated for this study are available on request to the corresponding author.

## Ethics Statement

All animal experiments were approved by the Institutional Animal Care and Use Committee (IACUC) at Augusta University and were performed in adherence to the NIH Guidelines on the Use of Laboratory Animals. Male Sprague Dawley rats of ages 10–12 weeks were obtained from Charles River Laboratory (Wilmington, MA, USA).

## Author Contributions

XC designed, performed, and interpreted most of the experiments. RR took part in conceptualization, planning, interpretation of the data, and edited and finalized the manuscript. RS and MD participated in the interpretation of the data, editing, and finalizing the manuscript.

### Conflict of Interest

The authors declare that the research was conducted in the absence of any commercial or financial relationships that could be construed as a potential conflict of interest.
